# Impact of hearing aid use on cognitive function in elderly individuals with hearing loss: a prospective study

**DOI:** 10.1016/j.bjorl.2026.101785

**Published:** 2026-04-16

**Authors:** Henrique Cannever Velho, Murilo Aparecido Sanches Cruz Schichi, Tyuana Sandim da Silveira Sassi, Jerusa Roberta Massola de Oliveira, Rubens Neto, Luiz Fernando Manzoni Lourençone

**Affiliations:** a Faculdade de Medicina da Universidade de São Paulo, São Paulo, SP, Brazil; b Faculdade de Medicina de Bauru da Universidade de São Paulo, Bauru, SP, Brazil; c Hospital de Reabilitação de Anomalias Craniofaciais da Universidade de São Paulo, Bauru, SP, Brazil

**Keywords:** Presbycusis, Hearing aids, Cognition

## Abstract

**Objective:**

To evaluate the cognition and the depressive symptoms of elderly individuals with hearing impairment before and after the use of Hearing Aids (HA), in order to determine whether auditory rehabilitation can influence cognition and mood.

**Methods:**

This is a prospective longitudinal study including elderly participants (>60 years) treated at the Hospital for Rehabilitation of Craniofacial Anomalies (HRAC), University of São Paulo (USP), who had indication for the use of hearing aids for auditory rehabilitation. Prior to HA fitting, tests were applied to assess cognition (Mini-Mental State Exam, verbal fluency test, and clock drawing test), mood (Geriatric Depression Scale), and restriction in daily life activities due to hearing loss (HHIE). These tests were repeated at 6- and 12-months after HA fitting.

**Results:**

The study included 56 elderly individuals with a mean age of 73.9-years (±6.9). After 6-months of HA use, a significant increase was observed in the MMSE score ‒ from 23.4 (±4.8) to 24.8 (±4.5) (p = 0.016) ‒ and in the clock test score ‒ from 5.7 (±3.7) to 7.0 (±3.0) (p = 0.016). Participants without initial cognitive decline showed no significant differences. Among those re-evaluated after 12-months, the MMSE score increased from 25.6 (±3.0) to 28.2 (±1.7) (p = 0.001). Verbal fluency improved from 11.0 (±2.7) to 14.3 (±4.2) (p = 0.0013), and the clock test from 7.5 (±2.1) to 8.5 (±1.3) (p = 0.0387). In the subgroup without initial cognitive decline, only verbal fluency showed a statistically significant improvement (p = 0.0031).

**Conclusion:**

Auditory rehabilitation through HA fitting had a positive impact on cognition in elderly individuals with hearing loss, especially in those who present cognitive decline in the initial tests.

**Level of evidence:**

According to the Oxford Centre for Evidence-Based Medicine 2011 Levels of Evidence, the present study corresponds to Level 3 evidence, being a non-randomized prospective longitudinal study with pre and post intervention assessments.

## Highlights

• Auditory stimulation may modulate cognitive function in older adults.
• Cognitive scores improved after 6- and 12-months of hearing aid use.
• Greater cognitive benefits were observed in participants with prior decline.
• Verbal fluency and visuospatial skills showed significant post-HA gains.
• Hearing aids reduced self-perceived auditory handicap over time.

## Introduction

Nowadays, many countries are undergoing demographic transition, with a decreasing proportion of young individuals and an increasing proportion of older adults in the population.^1^ Longevity has a significant impact in the elderly’s quality of life, since advancing age is usually followed by declines in physical, psychological, and social functioning.^2^

One system that is commonly affected by aging is the sensory system. Among sensory deficits, hearing loss is especially prevalent in the elderly. It is estimated that by the age of 60, 44% of individuals experience significant hearing loss; between 70- and 79-years, this number increases to 66%, and after 80, to 90%.^3^

Age-related hearing loss stems from changes in both the peripheral and central auditory systems. Peripheral alterations include loss of outer hair cells in the cochlea, cochlear neuron loss, and strial degeneration. Central changes involve degeneration of neurons in the vestibulocochlear nerve, the medial and superior olivary complex, and the cochlear nucleus. These contribute to reduced speech understanding, auditory processing, and sound localization.^4^

Presbycusis, the age-related hearing loss, is the leading cause of hearing impairment in the elderly population.^3^ It is marked by bilateral, symmetric loss ‒ more pronounced at high frequencies ‒, diminished comprehension (especially in noisy environments), reduced central auditory processing, and impaired sound source localization.^5^ Presbycusis usually starts by defects in the peripheral auditory system, but there are evidences indicating that this peripheral deficit could lead to alterations in the central system, contributing to hearing loss.^4^

Hearing loss has been linked not only to reduced quality of life, but also to accelerated and persistent cognitive decline in the elderly,^7^ affecting multiple cognitive abilities, such as memory, processing speed, attention and visuospatial orientation.^8^ Individuals with severe hearing loss are at higher risk for cognitive deterioration than those with mild/moderate loss.^7^ Additionally, hearing-impaired individuals have a 94% higher risk of dementia compared to those with normal hearing.^9^

There are many theories that attempt to explain the link between hearing loss and dementia,^10^ and understanding these mechanisms is vital for developing effective prevention and intervention strategies.^11^ The “cascade theory” suggests that reduced peripheral auditory input leads to decreased neural stimulation in the auditory cortex, causing synaptic loss and cortical atrophy, which may result in cognitive decline.^10^ If this hypothesis holds true, it could mean that auditory rehabilitation could mitigate or even prevent cognitive decline.^6^

There are studies suggesting that hearing rehabilitation in the elderly can reduce cognitive decline and improve overall quality of life,^12^ as well as general, social and emotional health.^6^ However, there is no consensus in literature about that,^12^, ^13^, ^14^ which emphasizes the need for further research into how auditory stimulation influences cognition and life quality in older adults presenting hearing loss.

Hearing aids are typically the first choice for rehabilitation in elderly individuals with mild to moderate hearing loss. For profound hearing deficits, cochlear implants may be considered.^3^ Hearing aids amplify sounds at affected frequencies and deliver them to the middle ear. When appropriate, binaural fitting offers several benefits, including improved sound localization, spatial balance, better signal-to-noise ratio, discrimination, and central auditory stimulation.^15^

This study aims to evaluate the cognitive function of elderly individuals before and after HA fitting, to determine whether auditory rehabilitation through acoustic stimulation can positively influence cognitive function.

## Methods

This was a prospective, longitudinal study involving elderly participants with hearing loss who were followed at the Hospital for Rehabilitation of Craniofacial Anomalies (HRAC), University of São Paulo (USP), and had the medical indication for the use of Hearing Aids (HA) as part of their auditory rehabilitation. The study was approved by the Research Ethics Committee for Human Beings of HRAC-USP.

Inclusion criteria were individuals aged 60 or older, of any gender and any socioeconomic status or scholarity, presenting bilateral sensorineural hearing loss, who underwent the selection and fitting process for hearing aids at HRAC-USP. Participants were fitted with different types of hearing aids, including Behind-The-Ear (BTE), In-The-Ear (ITE), and Open-Fit (OFH) devices. The choice and selection of the amplification device followed guidelines from Audiology Societies, which recommend programming based on audiological results and electroacoustic characteristics of the hearing aid (e.g., gain, maximum output), applying prescriptive rules appropriate for the elderly population. Exclusion criteria included individuals who had previously undergone any hearing treatment or who had a medical diagnosis of dementia, significant other sensory deficits or any type of hearing loss other than bilateral sensorineural hearing loss. Although many countries define elderly individuals as those aged 65-years or older,^16^ the Brazilian Statute of the Elderly establishes this threshold at 60-years.^17^ Given that the present study was conducted in Brazil, it was chosen to adopt the definition provided by the national Statute of the Elderly.

Participants attending the hearing aid selection and fitting unit were invited to participate in the study through a written informed consent form, which explained all necessary details. All participants listed here gave informed consent.

Before HA fitting, participants were evaluated for cognitive function, mood, and participation restrictions in daily activities due to hearing loss. These tests were repeated at 6- and 12-months post-fitting.

### Cognitive assessment

Three different cognitive tests were used: the Mini-Mental State Examination (MMSE), the Verbal Fluency Test (VFT) and the Clock Drawing Test (CDT)

### Mini-Mental State Examination (MMSE)

The MMSE is the most widely used cognitive screening tool worldwide, with sensitivity and specificity of 81% and 89%, respectively.^18^ It takes 5–10 min to administer and evaluates several cognitive domains: spatial and temporal orientation, immediate and delayed recall, calculation, language, repetition, comprehension, writing, and copying a drawing. Scores range from 0 to 30. Cut-off scores vary by education level: 19 for illiterates; 23 for 3 years of education; 24 for 4–7 years; and 28 for more than 7-years. Scores below these suggest cognitive decline.^19^

### Verbal fluency test

The Verbal Fluency Test assesses memory and language. The patient was asked to name as many animals as possible in one minute. The score was based on the number of correct words recalled.^20^

### Clock drawing test

The patient was asked to draw a clock showing the time “two forty-five”. The drawing was evaluated for accuracy in placing numbers and hands, with scores ranging from 0 to 10.

### Assessment of depressive symptoms

To assess depressive symptoms, the 15-item version of the Geriatric Depression Scale (GDS-15) was used. This is the most popular instrument used to evaluate depressive symptoms in the elderly and it was developed specifically for this population. It consists of yes/no questions administered as an interview. A score of 5 or more suggests depression.^21^

### Assessment of hearing participation restriction

The Hearing Handicap Inventory for the Elderly-Screening (HHIE-S), a 10-item questionnaire, was used to assess the self-perceived psychosocial effects of hearing loss. Five items address emotional consequences, and five focus on social and situational impacts. Responses are “yes”, “sometimes”, or “no”. Scores range from 0 to 40, with higher scores indicating greater perceived handicap.^22^

### Statistical analysis

Paired-samples *t*-test were conducted to assess changes in scores of the above described tests between baseline and 6- and 12-month follow-up. Normality of the data was assessed using the Shapiro–Wilk test, and assumptions were met.

## Results

Fifty-six elderly individuals participated in the study, with a mean age of 73.9-years (±6.9), 24 of whom were male (42.9%) and 32 female (57.1%). Of the 56 participants, 7 (12.5%) had no education, 23 (41.1%) had studied for 1 to 4-years, 14 (25%) had studied for 5 to 8-years and 12 (21.4%) had studied for more than 8-years (Table 1).

**Table 1:** General results.

Group	Number of patients	Sex (M/F)	Average age (±DP)	Meem (±DP)	GDS (±DP)	FVT (±DP)	CDT (±DP)	HHIE (±DP)
**Before HA**	56	24/32 (42.9%/57.1%)	73.9 ± 6.9	23.4 ± 4.3 (9–29)	4.9 ± 3.5 (0–15)	11.3 ± 3.1 (4–19)	6.5 ± 3.3 (0–10)	20.25 ± 11.3
6-months								
Before HA	27	14/13 (51.9%/48.1%)	72.9 ± 6.6	23.4 ± 4.8(9–29)	4.9 ± 3.6 (0–15)	10.8 ± 3.4 (4–19)	5.7 ± 3.7 (0–10)	19.3 ± 12.2
6-months				24.8 ± 4.5 (19–30)	4.3 ± 3.2 (0–15)	12.0 ± 4.7 (5–20)	7.0 ± 3.0 (0–10)	12.4 ± 12
12-months								
Before HA	19	7/12 (36.8%/63.2%)	72.3 ± 5.3	25.6 ± 3.0 (18–29)	3.8 ± 2.7 (0–10)	11 ± 2.7 (6–15)	7.5 ± 2.1 (4–9)	16.2 ± 11.6
12-months				28.2 ± 1.7 (25–30)	3.5 ± 2.4 (0–10)	14.3 ± 4.2 (6–27)	8.5 ± 1.3 (4–10)	9.7 ± 9.1

The initial average score on the Mini-Mental State Examination (MMSE) was 23.4 (±4.3), and on the Geriatric Depression Scale (GDS) it was 4.9 (±3.5). The other tests applied showed the following average scores: verbal fluency 11.3 (±3.1), clock drawing test 6.5 (±3.3), and HHIE total 20.2 (±11.3).

**Fig. 1:**
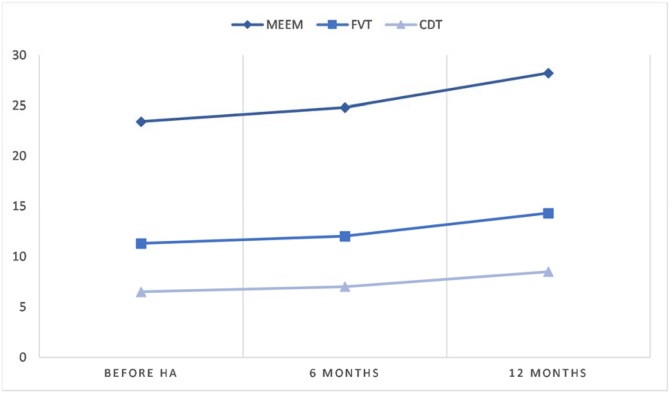
Increase of cognitive tests score through time.

**Fig. 2:**
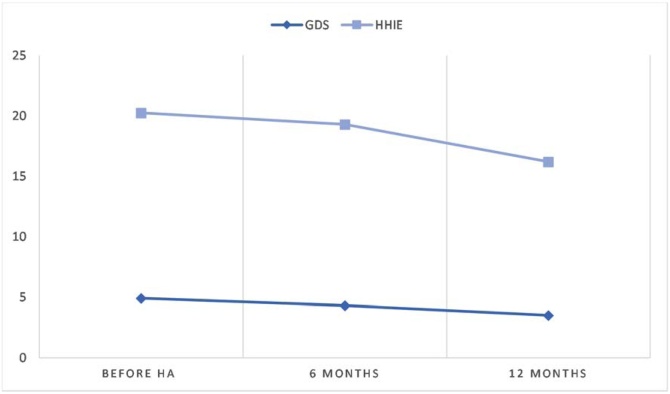
Decline of GDS and HHIE scores through time.

Of the 56 participants, 27 (48.2%) underwent reevaluation after 6-months of use of Hearing Aids (HA) and 19 (33.9%) after 12-months. All 56 participants were invited to repeat the tests at both time points, but only a subset attended, explaining the reduced sample size over time. Due to this, analyses were not performed between different educational levels, which is a limitation of this study. The mean score of each test through time is illustrated in Fig. 1, Fig. 2.

### 6-months group (Table 2)

After 6-months of HA use, an average increase in the MMSE score was observed, from 23.4 (±4.8) to 24.8 (±4.5) (p = 0.016). The score on the clock drawing test also showed a significant improvement, from 5.7 (±3.7) to 7.0 (±3.0) (p = 0.016). HHIE scores decreased from 19.3 (±12.2) to 12.4 (±12.0) (p = 0.0157), indicating a lower perception of auditory restriction. Verbal fluency increased from 10.8 (±3.4) to 12.0 (±4.7), but without reaching statistical significance (p = 0.0808). The GDS score showed a slight reduction (4.9 to 4.3), without statistical significance (p = 0.484).

**Table 2:** 6-months group.

Group	Number of patients	Sex (M/F)	Average age (±DP)	Meem (±DP)	GDS (±DP)	FVT (±DP)	CDT (±DP)	HHIE (±DP)	Group	Number of patients
Cognitive decline −										
Before HA	12	8/4 (66.7%/ 33.3%)	71.9 ± 4.4	26.2 ± 3.2	4.9 ± 4.5	11.5 ± 1.8	7.5 ± 3.1	8.5 ± 5.5	4.9 ± 2.9	3.6 ± 3.1
6-months				25.7 ± 4.2	5.0 ± 4.0	13.7 ± 3.5	7.6 ± 2.4	7.9 ± 7.6	3.4 ± 4.1	4.5 ± 4.0
Cognitive decline +										
Before HA	15	6/9 (40.0%/ 60.0%)	73.6 ± 8.0	21.1 ± 4.7	4.8 ± 2.9	10.2 ± 4.2	4.3 ± 3.6	10.6 ± 6.6	5.2 ± 3.2	5.4 ± 3.7
6-months				24.1 ± 4.7	3.8 ± 2.5	10.9 ± 5.1	6.5 ± 3.4	4.9 ± 4.1	2.9 ± 2.7	2.0 ± 2.1

When stratifying participants according to the presence of initial cognitive decline, individuals with decline (n = 15) showed an average MMSE increase from 21.1 (±4.7) to 24.1 (±4.7) (p = 0.0005), while participants without decline (n = 12) showed no significant difference (p = 0.410).

### 12-months group (Table 3)

Among participants reevaluated after 12-months of HA use, there was a significant increase in the average MMSE score from 25.6 (±3.0) to 28.2 (±1.7) (p = 0.001). Verbal fluency improved from 11.0 (±2.7) to 14.3 (±4.2) (p = 0.0013), and the clock drawing test improved from 7.5 (±2.1) to 8.5 (±1.3) (p = 0.0387). The HHIE score decreased from 16.2 (±11.6) to 9.7 (±9.1) (p = 0.0077), reflecting a lower perception of the impact of hearing impairment on daily life. The GDS score changed from 3.8 (±2.7) to 3.5 (±2.4), without statistical difference (p = 0.675).

**Table 3:** 12-months group.

Group	Number of patients	Sex (M/F)	Average age (±DP)	Meem (±DP)	GDS (±DP)	FVT (±DP)	CDT (±DP)	HHIE (±DP)	Group	Number of patients
Cognitive decline −										
Before HA	12	6/6 (50%/50%)	73.2 ± 4.4	26.8 ± 2.5	3.8 ± 3.2	11.8 ± 1.8	8.5 ± 1.4	13.2 ± 8.9	8.3 ± 4.7	5.3 ± 5.3
12-months				27.9 ± 1.8	3.1 ± 2.2	13.9 ± 1.9	8.5 ± 1.6	8.7 ± 7.4	6 ± 4.8	3.5 ± 4.4
Cognitive decline +										
Before HA	7	1/6 (14.3%/ 85.7%)	70.6 ± 6.2	23.6 ± 3.0	3.9 ± 1.7	9.9 ± 3.7	5.9 ± 2.0	10.7 ± 7.3	5.6 ± 3.4	5.1 ± 4.1
12-months				28.6 ± 1.6	4.1 ± 2.7	15.0 ± 6.8	8.5 ± 0.5	5.7 ± 5.9	3.0 ± 3.1	2.7±.3.0

In participants with initial cognitive decline (n = 7), the MMSE increased significantly from 23.6 (±3.0) to 28.6 (±1.6) (p = 0.0016). Verbal fluency also showed significant improvement (p = 0.0435), as did the clock drawing test (p = 0.0335). In the subgroup without cognitive decline (n = 12), only verbal fluency demonstrated statistically significant improvement (p = 0.0031).

### Correlation between variables

Statistically significant correlations were observed between MMSE scores and verbal fluency (p = 0.003), and with the clock drawing test (p < 0.001), both at 6- and 12-months. The HHIE also showed a significant correlation with the MMSE in the first evaluation (p = 0.049).

## Discussion

The results of this study demonstrated a significant improvement in general cognitive functions, especially in the scores of the Mini-Mental State Examination (MMSE), verbal fluency, and performance on the clock drawing test, after 6- and 12-months of auditory rehabilitation with Hearing Aids (HA) in elderly individuals with hearing loss. Additionally, there was a marked reduction in the self-perception of the impact of hearing impairment on daily life, measured by the HHIE. These findings support the hypothesis that sound stimulation plays a relevant role in modulating cognitive function in older adults, particularly in those with initial cognitive decline.

The average increase in MMSE scores from 23.4 to 24.8 points at 6-months, and from 25.6 to 28.2 points at 12-months, reflects sustained improvement in global cognition, with statistical significance. This improvement was more pronounced in the subgroup of patients with previous cognitive decline, reinforcing the idea that auditory rehabilitation has a greater impact on individuals with compromised cognitive function ‒ a finding consistent with Gurgel et al. (2022), who demonstrated cognitive gains after cochlear implant adaptation in elderly patients with MMSE ≤24-points^23^ and in line with the metanalysis of Yang (2022)^24^ that found no statistically significant effect of hearing aid use on cognitive function of subjects without dementia. Although the increase in the MMSE scores indicates there is a cognition benefit on auditory rehabilitation in the elderly with hearing loss, the clinical relevance of this finding was not assessed in this study.

The improvement in verbal fluency is also noteworthy, with significant growth after 12-months and a positive trend at 6-months. Considering that verbal fluency tasks require sustained attention, processing speed, and semantic memory, the recovery of these skills after HA use reinforces the cognitive load theory, according to which reduced auditory effort allows for the redistribution of cognitive resources to more complex tasks.^25^

Additionally, performance on the clock drawing test, associated with executive functions and visuospatial integration, also improved significantly, which is consistent with the findings of Mosnier et al. in elderly individuals with profound hearing loss who received cochlear implants and showed improvement in attention and nonverbal memory tests following auditory intervention.^26^ These associations between the use of hearing aids devices and the improvement in cognitive test scores are in accordance with what has been shown in literature until now,^27^ which could mean that treating hearing loss could correct a certain degree of cognitive decline.

The lack of statistically significant improvement in Geriatric Depression Scale (GDS) scores should be interpreted with caution. Although there was no global change in mood, participants had relatively low initial scores, which may have limited the detection of change. On the other hand, the significant reduction in HHIE scores indicates a positive perception of rehabilitation in emotional and social aspects of hearing, which may indirectly act as a protective factor against isolation and apathy, known predictors of cognitive decline and depression.^28^, ^29^, ^30^

It is important to highlight that the ACHIEVE study, the largest randomized clinical trial on the subject to date, did not find a significant difference in the progression of global cognitive decline between groups with and without hearing intervention over three years.^25^ However, a stratified analysis revealed that the beneficial effects were concentrated in subgroups at increased risk for dementia ‒ a finding that aligns directly with the stratification performed in this study, where only patients with initial cognitive decline showed significant gains in MMSE and other tests.

These data reinforce the notion that auditory rehabilitation does not act uniformly on cognitive aging, being more effective when applied to vulnerable individuals or those already in decline. The interaction between hearing and cognition, therefore, is not only correlational but possibly causal in certain contexts, as argued by Dawes et al.,^31^ who emphasize hearing as a critical mediator of continuous cognitive stimulation and social engagement in older adults.^23^

Finally, the results presented here support the auditory-cognitive cascade theory,^10^ which states that peripheral hearing loss triggers central changes due to reduced auditory input, leading to cortical reorganization and, eventually, functional cognitive decline. Auditory rehabilitation, by restoring part of this input, could interrupt or partially reverse this process, as proposed by Uchida et al.

## Conclusion

The use of hearing aids for auditory rehabilitation demonstrated a positive impact on the cognitive function of elderly patients with hearing loss. Improvements were observed in Mini-Mental State Examination, Verbal Fluency Test, Clock Drawing Test and self-perceived auditory handicap, especially among individuals with initial cognitive decline. These findings support the hypothesis that stimulation through hearing aid use can modulate or delay cognitive deterioration in aging. Although no substantial effect on depressive symptoms was observed, the results highlight the broader benefits of auditory rehabilitation on cognitive health and functional aging, emphasizing its importance in geriatric and audiological clinical practice.

## ORCID ID

Henrique Cannever Velho: 0000-0002-8306-7272

Murilo Aparecido Sanches Cruz Schichi: 0009-0008-6790-4908

Tyuana Sandim da Silveira Sassi: 0000-0001-5303-3672

Jerusa Roberta Massola de Oliveira: 0000-0001-8771-3588

Rubens Neto: 0000-0001-5313-5214

## Funding

This research did not receive any specific grant from funding agencies in the public, comercial or not-for-profit sectors.

## Data availability statement

The authors declare that all data are available in repository.

## Conflicts of interest

The authors declare no conflicts of interest.
